# HrpE, the major component of the Xanthomonas type three protein secretion pilus, elicits plant immunity responses

**DOI:** 10.1038/s41598-018-27869-1

**Published:** 2018-06-29

**Authors:** Natalia Gottig, Cecilia V. Vranych, Germán G. Sgro, Ainelén Piazza, Jorgelina Ottado

**Affiliations:** Instituto de Biología Molecular y Celular de Rosario, Consejo Nacional de Investigaciones Científicas y Técnicas (IBR-CONICET) and Facultad de Ciencias Bioquímicas y Farmacéuticas, Universidad Nacional de Rosario (UNR). Ocampo y Esmeralda, Rosario, Argentina

## Abstract

Like several pathogenic bacteria, Xanthomonas infect host plants through the secretion of effector proteins by the Hrp pilus of the Type Three Protein Secretion System (T3SS). HrpE protein was identified as the major structural component of this pilus. Here, using the *Xanthomonas citri* subsp. *citri* (*Xcc*) HrpE as a model, a novel role for this protein as an elicitor of plant defense responses was found. HrpE triggers defense responses in host and non-host plants revealed by the development of plant lesions, callose deposition, hydrogen peroxide production and increase in the expression levels of genes related to plant defense responses. Moreover, pre-infiltration of citrus or tomato leaves with HrpE impairs later Xanthomonas infections. Particularly, HrpE C-terminal region, conserved among Xanthomonas species, was sufficient to elicit these responses. HrpE was able to interact with plant Glycine-Rich Proteins from citrus (CsGRP) and Arabidopsis (AtGRP-3). Moreover, an Arabidopsis a*tgrp-3* knockout mutant lost the capacity to respond to HrpE. This work demonstrate that plants can recognize the conserved C-terminal region of the T3SS pilus HrpE protein as a danger signal to defend themselves against Xanthomonas, triggering defense responses that may be mediated by GRPs.

## Introduction

Phytopathogens of the Xanthomonas genus are bacteria that produce devastating diseases in a diversity of mono- and dicotyledonous plants worldwide, comprising important crop plants^1^. Like animal pathogenic bacteria, these plant pathogens colonize their hosts through the secretion of bacterial virulence factors into the extracellular milieu or directly into the host cell. The Type Three Secretion System (T3SS) is a conserved trans-envelope multiprotein complex that delivers bacterial effector proteins across three membranes into the cytosol of eukaryotic cells^2,3^. The T3SS spans both bacterial membranes through membrane-associated ring structures that enclose an inner transport channel and is associated with an extracellular filamentous appendage, termed ‘needle’ in animal pathogens and extracellular Hrp pilus in plant pathogens^3^. This structure serves as a conduit through which substrates are transported to the host pathogen interface and into the host cell cytosol. Plant-pathogenic bacteria carrying mutations in genes encoding the T3SS neither elicit Hypersensitive Response (HR), a plant defense response that restricts bacterial growth in resistant plants, nor cause pathogenicity in susceptible host plants. Therefore these genes are referred as HR and pathogenicity (*hrp*) genes^4^. A major role in pathogenicity and HR^5^, and modulation of bacterial biofilm formation^6^, were established for the T3SS in *Xanthomonas citri* subsp. *citri* (*Xcc*), the phytopathogen causing citrus canker^7^. *Xcc* contains a gene in the *hrp* cluster, named *hrpE*, which encodes the 9.7-kDa HrpE protein. *Xcc* HrpE denotes a high degree of conservation with HrpE proteins present in other *Xanthomonas* such as *X*. *campestris* pv. *vesicatoria*, *X*. *oryzae* pv. *oryzae*, *X*. *axonopodis* pv. *glycines* and *X*. *campestris* pv. *campestris*, especially in the C-terminal region^8^. This protein was identified as the main structural component of the Hrp pilus in *Xanthomonas campestris* pv. *vesicatoria*^8^.

Plants have evolved the capacity to recognize some molecules (known as Pathogen-Associated Molecular Patterns, PAMPs) highly conserved within a class of microbes that have an essential function in microbial fitness or survival. Through cell surface receptors called Pattern Recognition Receptors (PRRs), plants recognize PAMPs as non-self molecules and subsequently activate the PAMP-triggered defense response that restricts pathogen growth and thus hampers tissue colonization^9^. This defense response includes the induction of defense-associated genes, callose deposition and oxidative burst. Examples of bacterial PAMPs include flagellin, elongation factor Tu, lipopolysaccharide, peptidoglycans and methylated DNA fragments^10^. In *Xcc*, a harpin protein^11^, a non-fimbrial adhesin^12^ and the osmoprotectant sugar trehalose^13^, also have a role in the elicitation of plant defense responses. In view of the high degree of conservation of HrpE in the Xanthomonas genus and since HrpE is the major component of the HrpE pilus which is crucial for pathogenicity and is exposed on the bacterial surface, it had been previously hypothesized that HrpE would be a candidate to act as a PAMP^14^. However, there are no reports stuying this role for HrpE or indicating if plants may have evolved mechanisms to recognize this protein in order to detect pathogen attack at an early stage and encourage a defense response. Here, the functional characterization of *Xcc* HrpE as an elicitor of plant defense responses was investigated. Infiltration of pure recombinant HrpE showed that this protein is able to elicit HR responses and to increase known markers for PAMP-triggered immunity such as callose deposition, hydrogen peroxide production and the expression of plant defense genes^15^. The effect of HrpE in the induction of plant defense responses was corroborated by challenging HrpE-treated plants with virulent pathogens resulting in a strong outcome of plant resistance. Experiments employing HrpE fragments showed that the most conserved C-terminal region is responsible for triggering plant responses. Moreover, plant proteins that can recognize HrpE and mediate HrpE-triggered plant defense responses were identified. Particularly, citrus and Arabidopsis Glycine-Rich Protein (GRP), named CsGRP and AtGRP-3, were able to interact with HrpE. Moreover, HrpE lost the capacity to elicit defense responses in a*tgrp-3* knockout mutant Arabidopsis plants. This is the first report of a plant protein involved in direct recognition of a Xanthomonas Hrp pilus protein, with a role in signaling mechanisms mediating plant responses against pathogens.

## Results

### HrpE induces defense responses in host and non-host plants

**Figure 1:**
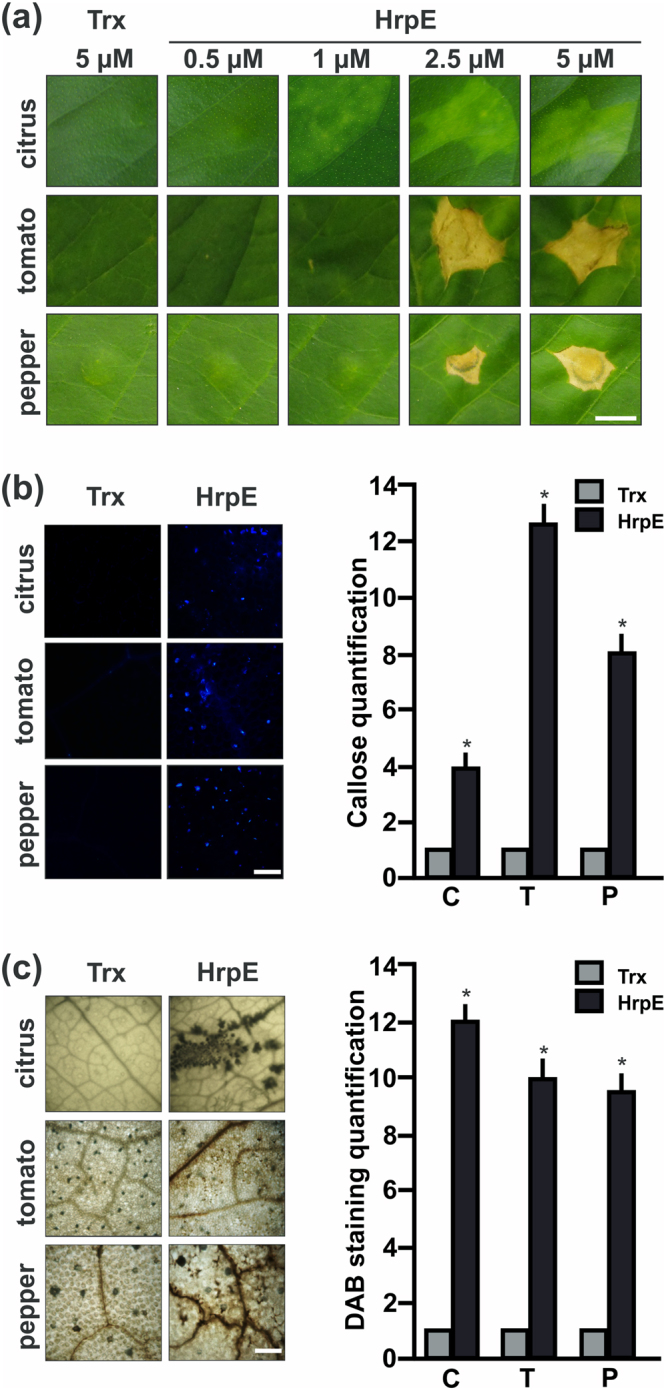
Analysis of citrus, tomato and pepper leaves responses to *Xcc* HrpE. (**a**) Representative photographs of leaves responses to the infiltration of pure HrpE-Trx-^6^His (HrpE), ranging from 0.5 µM to 5 µM, and 5 µM Trx-^6^His (Trx) (control) 1 dpi. Bar indicates 0.5 cm. (**b**) Representative fluorescence microscopy photographs of aniline blue staining of callose deposition in leaves infiltrated with 2.5 µM HrpE and Trx (control) 8 hpi (tomato and pepper) and 16 hpi (citrus). Bar indicates 20 μm. The right panel shows the quantification of callose intensities in citrus (C), tomato (T) and pepper (P) tissues infiltrated with HrpE (black bars) relative to Trx (grey bars). (**c**) Representative photographs of DAB stained leaves infiltrated as in (**b**) (Bar indicates 1 mm). In citrus, H_2_O_2_ production is observed as brown precipitates in leaf tissues and in tomato and pepper, the brown precipitates are observed near to the leaf veins. The right panel shows the quantification of DAB staining in infiltrated C, T and P tissues with HrpE (black bars) relative to Trx (grey bars). For both, callose and DAB intensities quantifications, the means were calculated from 25 photographs obtained from different treated leaves from three independent experiments. Error bars indicate standard deviations. Asterisks represent significant differences based on one-way ANOVA (*p* < *0*.*05*).

The role of HrpE as an elicitor of plant defense responses was analyzed by infiltrating the purified HrpE-Trx-^6^His (HrpE) protein at different concentrations into *Citrus sinensis* (citrus) *Xcc* host and non-host plants, such as *Solanum lycopersicum* (tomato) and *Capsicum annuum* (pepper). HrpE could be expressed and purified only as a fusion protein to thioredoxin, because of its lability during the purification process. Hence, Trx-^6^His (Trx) was purified in the same conditions as HrpE and used as a control. The degree of purity and the integrity of proteins preparations was corroborated by SDS-PAGE analysis (Fig. S1). One day post-infiltration (dpi), HrpE infiltrated at concentrations of 1, 2.5 and 5 µM caused visible responses in citrus leaves with the intensity of the chlorotic lesions increasing in a dose-dependent manner while leaves infiltrated with 5 µM Trx showed no lesions (Fig. 1a). The infiltration of non-host leaves with 2.5 and 5 µM HrpE produced necrotic lesions (Fig. 1a). In addition, callose deposition, a known marker for plant defense responses, was evaluated in HrpE-infiltrated citrus and non-host leaves. Callose deposits staining was performed with aniline blue and quantified 8 hours post-infiltration (hpi) in tomato and pepper, and 16 hpi in citrus. HrpE induced significant callose deposition in all the plants assayed (*p* < *0*.*05*) while control leaves infiltrated with Trx showed no callose deposition (Fig. 1b). Finally, H_2_O_2_ levels, assessed by staining with DAB, were significantly higher in HrpE infiltrated-leaves than in Trx infiltrated controls (Fig. 1c).

### HrpE increases the expression of genes related to plant defense responses

**Figure 2:**
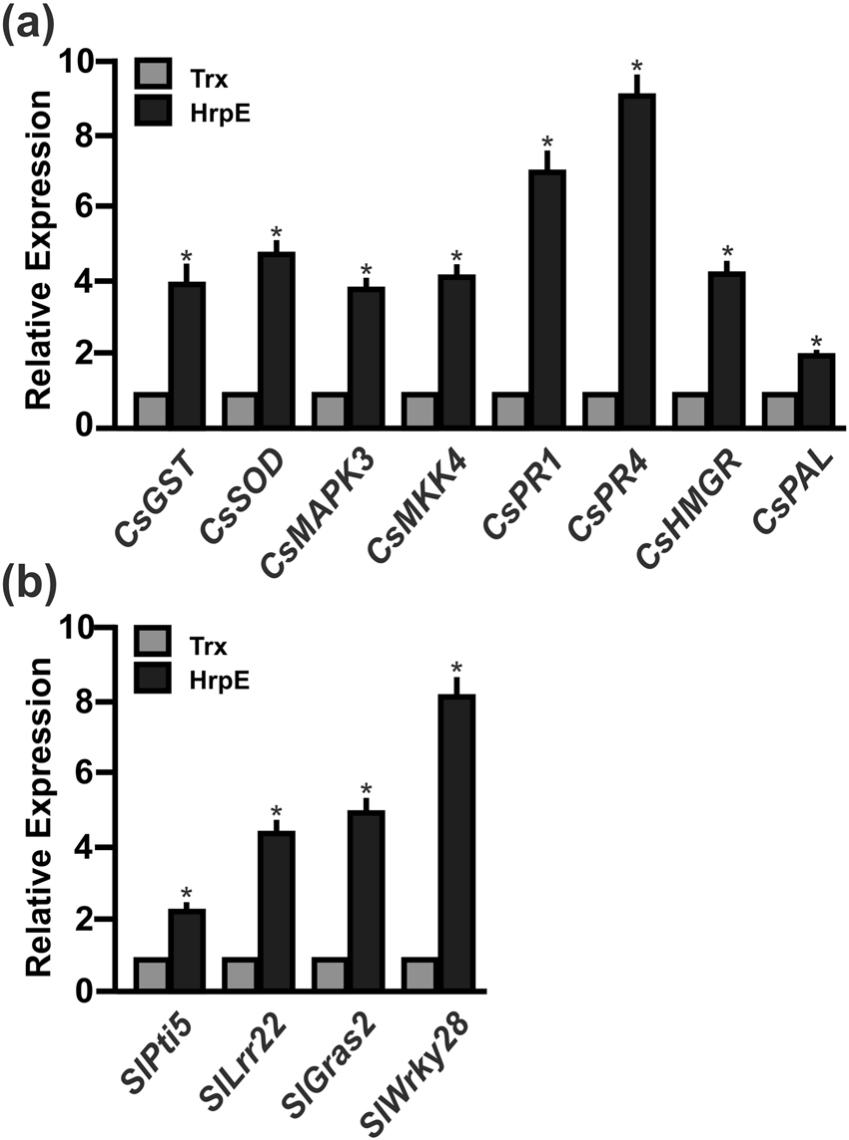
Analysis of the expression levels of citrus and tomato defense response-related genes in leaves infiltrated with HrpE by qRT-PCR. (**a**) Expression levels of defense response-related genes in citrus leaves, 16 hpi, or (**b**) in tomato leaves, 4 hpi, with 2.5 µM HrpE. Black bars indicate the expression levels of the genes relative to the expression levels of 2.5 µM Trx infiltrated controls (grey bars). Values are the means of four biological replicates with three technical replicates each. Error bars indicate standard deviations. Results were analyzed by one-way ANOVA (*p* < *0*.*05*). Asterisks represent significant differences based on one-way ANOVA (*p* < *0*.*05*).

The expression of genes involved in plant defense responses was analyzed in citrus and tomato, as examples of compatible and incompatible interactions, respectively. For this purpose, leaves were infiltrated with HrpE or with the Trx control. Plant RNA from infiltrated citrus (1, 8 and 16 hpi) and tomato (1 and 4 hpi) tissues was extracted and qRT-PCR were performed. In citrus leaves, several genes markers of plant defense responses^13^ were analyzed: Glutathione-S-Transferase (*CsGST*), Superoxide Dismutase (*CsSOD*), Mitogen Activated Protein Kinase 3 (*CsMAPK3*), Mitogen Activated Protein Kinase Kinase 4 (*CsMKK4*), Pathogenesis Related 1 and 4 (*CsPR1* and *CsPR4*), 3-Hydroxy-Methylglutaryl CoA Reductase (*CsHMGR*) and Phenylalanine Ammonia Lyase (*CsPAL*). At 1 and 8 hpi with HrpE, the expression levels of all the analyzed genes were similar to that of the control leaves infiltrated with Trx-^6^His (data not shown). However, a significant increase in the expression levels of these genes was observed 16 hpi in tissues infiltrated with HrpE relative to the Trx control (*p* < *0*.*05*) (Fig. 2a). In tomato leaves, the expression levels of defense response-related genes^12^, including *SlPti5*, *SlLrr22* and *SlGras2*, and the transcription factor *SlWrky28*, were analyzed. A significant induction of the expression of all these genes was observed 4 hpi with HrpE relative to the Trx control (*p* < *0*.*05*) (Fig. 2b).

### Pre-treatment of citrus and tomato leaves with HrpE impairs *Xcc* and *Xcv* infections

**Figure 3:**
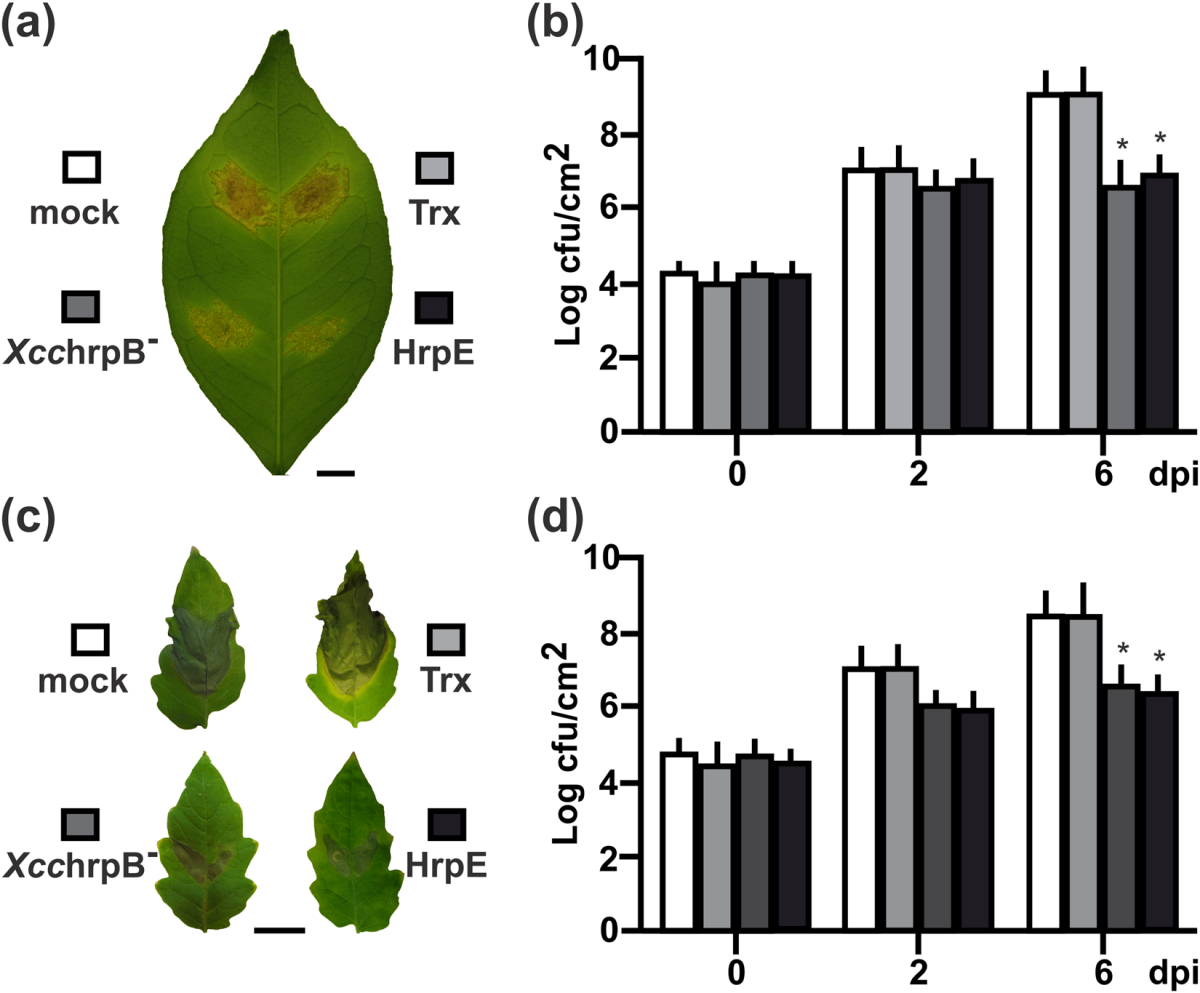
Analysis of bacterial infections in plants pre-infiltrated with HrpE. (**a**) Representative photographs of *Xcc* infected citrus and (**c**) *Xcv* infected tomato leaves that were pre-treated with 1 µM HrpE and *Xcc*hrpB^−^ (10^7^ cfu/mL); 1 µM Trx and mock (15 mM NaCl) were used as controls (Bar indicates 1 cm). (**b**,**d**) Quantification of bacterial growth in the leaves described above (**a** and **c**, respectively) at 0, 2 and 6 dpi. Values are the means obtained from 10 infiltrated leaves of each plant at different dpi. Error bars show the standard deviation. Asterisks indicate significant differences based on one-way ANOVA (*p* < *0*.*05*).

To further confirm that HrpE can promote plant defense responses, the potential of this protein to enhance disease resistance in citrus and tomato was investigated. Citrus and tomato leaves were pre-treated with 1 µM HrpE (a concentration that did not induce lesions, Fig. 1a). Controls included the pre-treatment with Trx (1 µM) or *Xcc*hrpB^−^ (10^7^ cfu/mL suspension), a mutant strain which does not produce infection symptoms but enhances plant defense responses, or mock infiltration (15 mM NaCl)^13^. Then, the pre-treated tissues were infiltrated with the virulent pathogens *Xcc* (citrus tissue, Fig. 3a) or *Xcv* (tomato tissue, Fig. 3c) at 10^6^ cfu/mL, and bacterial growth in plant tissue was monitored at different times post-infiltration. At 6 dpi, the population of *Xcc* was significantly (~100 times) lower (*p* < *0*.*05*) in the HrpE and *Xcc*hrpB^–^ pre-treated citrus leaves than in Trx treated or mock controls (Fig. 3b). Similar results were observed for tomato tissues, at 6 dpi (Fig. 3d).

### HrpE physically interacts with a citrus Glycine-Rich Protein *in vitro* and *in vivo*

**Figure 4:**
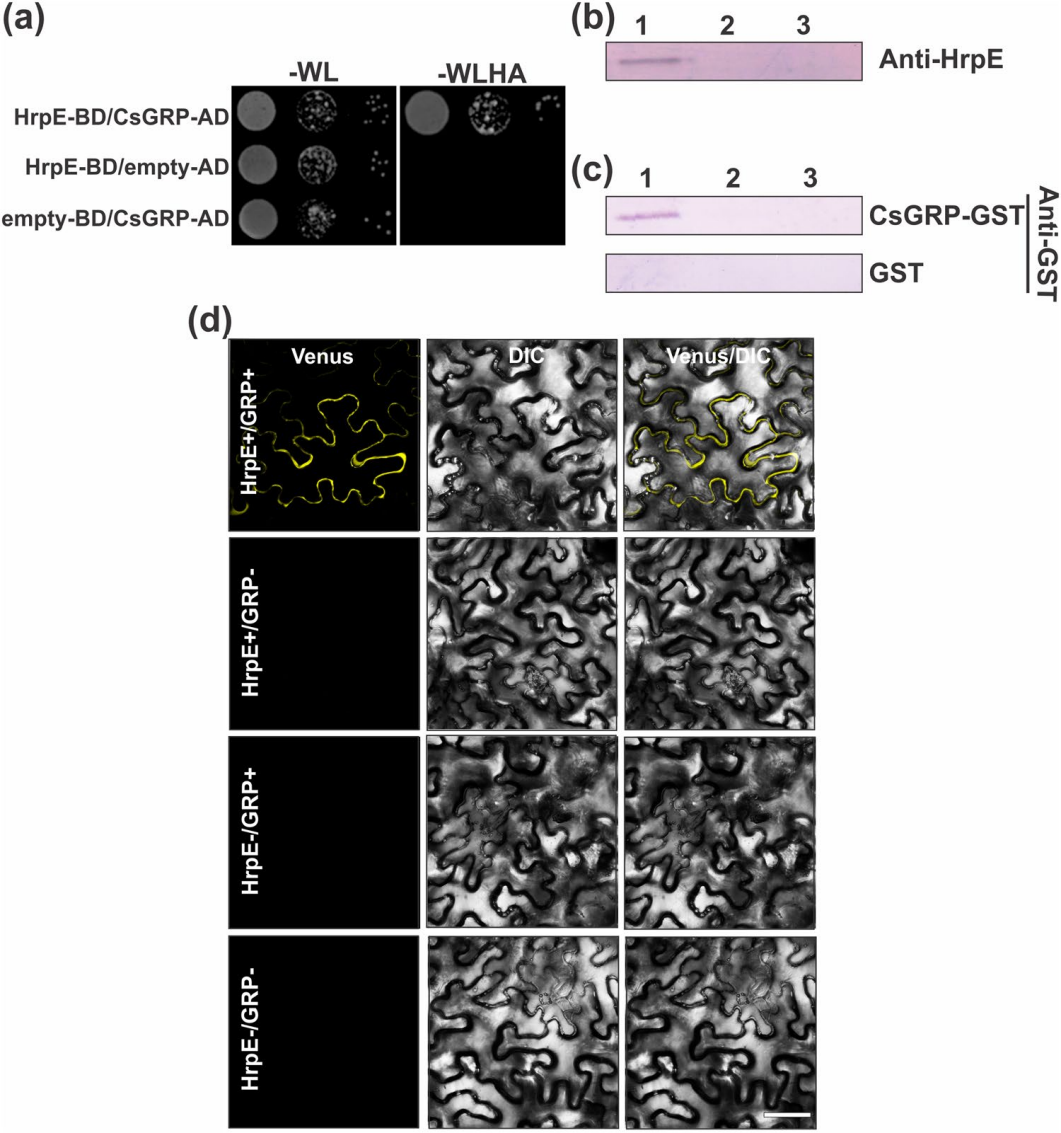
Yeast Two Hybrid, Far Western Blot and BiFC assays of the interaction between HrpE and CsGRP. (**a**) Representative photograph of YTH assay showing that HrpE interacts with CsGRP. Yeast cells were co-transformed with: HrpE-BD/CsGRP-AD, HrpE-BD/empty-AD and empty-BD/CsGRP-AD. Yeast growth (serial 1:10 dilutions) is shown in −WL plates and in −WLHA plates containing 35 mM 3AT. (**b**) Purification of the Hrp-pilus from *Xcc*hrpG^+^ (lane 1) and *Xcc*hrpG^−^ (lane 2) grown in XVM2 medium and from *Xcc*hrpG^+^ (lane 3) grown in SB. Proteins obtained from pilus preparations were analyzed by Tricine-SDS-PAGE and Western blot revealed with anti-HrpE polyclonal antibody. (**c**) Far Western blots showing interactions between HrpE present in the Hrp-pilus preparations and CsGRP-GST. Nitrocellulose membranes similar to that showed in (**b**) were incubated with 50 μg CsGRP-GST or GST as a control and, after washing, probed with anti-GST polyclonal antibody. (**d**) Confocal laser-scanning micrographs of the abaxial surface of *N*. *benthamiana* leaves. BiFC constructs of HrpE-nVenus/CsGRP-cCFP (HrpE+/GRP+), HrpE-nVenus/empty-cCFP (HrpE+/GRP−), empty-nVenus/GRP-cCFP (HrpE−/GRP+), and empty-nVenus/empty-cCFP (HrpE−/GRP−) were co-expressed in *N*. *benthamiana* using agroinfiltration. Scale bars represent 25 μm. DIC: Differential Interference Contrast.

To identify plant proteins directly interacting with HrpE, a Yeast Two-Hybrid (YTH) assay using HrpE as bait against a prey library derived from *C*. *sinensis* cDNA was performed. Among several putative HrpE interactors of particular relevance, the full-length coding region of a citrus Glycine-Rich Protein (CsGRP) (ID: XP_006469561.1) was identified (Table S1). To confirm whether HrpE and CsGRP directly interact, CsGRP was expressed in frame with the activation domain encoded by pGAL4 (pOAD), and HrpE was expressed in frame with the DNA binding domain encoded by pGAL4 (pOBD). These plasmids were co-transformed into *Saccharomyces cerevisiae* strain J694a and the interaction of HrpE with CsGRP (HrpE-BD/CsGRP-AD) was confirmed by the growth of yeast cells on medium lacking tryptophan, leucine, histidine and adenine (−WLHA) and supplemented with 35 mM of 3-amino-1,2,4-triazole (3AT) (Fig. 4a). HrpE-BD/empty-AD and empty-BD/CsGRP-AD transformations were used as controls and no yeast growth was observed in −WLHA selection medium (Fig. 4a). To confirm the results obtained with the YTH assay, the interaction between purified Hrp pilus and CsGRP was studied by another independent *in vitro* protein binding assay. Recombinant CsGRP was expressed and purified as a GST fusion protein. Then, pilus preparations from *Xcc*HrpG^+^ cells overexpressing T3SS proteins and, as control, from *Xcc*hrpG^−^ mutant cells that lack T3SS protein expression^16^, were obtained. Both strains were grown statically in XVM2 medium to induce the expression and assembly of the Hrp-pilus^17^. As an additional control, *Xcc*HrpG^+^ cells were grown in SB rich medium where Hrp-pilus formation is not induced. Pilus preparations were separated by Tricine-SDS-PAGE and Western blot analysis with an anti-HrpE polyclonal antibody revealed the presence of a unique band corresponding to the molecular weight of HrpE in the *Xcc*HrpG^+^ strain, grown in XVM2 medium (Figs 4b and S2). Far Western Blot assays were performed with all the pilus preparations and a positive interaction was observed between CsGRP and the Hrp pilus obtained from the *Xcc*HrpG^+^ strain grown in XVM2 medium (Figs 4c and S2).

Finally, to analyze HrpE/CsGRP interaction *in vivo*, Bimolecular Fluorescence Complementation (BiFC) assays were performed. HrpE was fused to the non-fluorescent N-terminal fragment of Venus (HrpE-nVenus) and CsGRP was fused to the non-fluorescent C-terminal fragment of cyan fluorescent protein (CsGRP-cCFP) in appropriate vectors to express the fusion proteins in *Nicotiana benthamiana*. Three days after HrpE-nVenus and CsGRP-cCFP were mixed and injected into *N*. *benthamiana* leaves, visible fluorescence was observed using confocal microscopy, suggesting a specific interaction between HrpE and CsGRP (Fig. 4d). No fluorescence was detected after the expression of HrpE-nVenus/empty-cCFP, empty-nVenus/GRP-cCFP and empty-nVenus/empty-cCFP controls (Fig. 4d). The HrpE-CsGRP interaction showed a pattern of fluorescence indicative of localization at the cell periphery, probably in the apoplast (Fig. 4d). Plasmolysis of this tissue showed a strong fluorescence signal not associated with the retracted membranes, suggesting that the interaction does not take place at the plasma membrane (Figs S3a and b). As a positive control of plasmolysis the confocal microscopy analysis was performed with AvrXacE2-GFP protein that localized at the plasma membrane^18^ and (Fig. S3c) and upon plasmolysis its fluorescence was associated with some retracted area of the plasma membrane (Fig. S3d).

### HrpE interacts with AtGRP-3 and *AtGRP-3* mutant plants responded differently to HrpE

**Figure 5:**
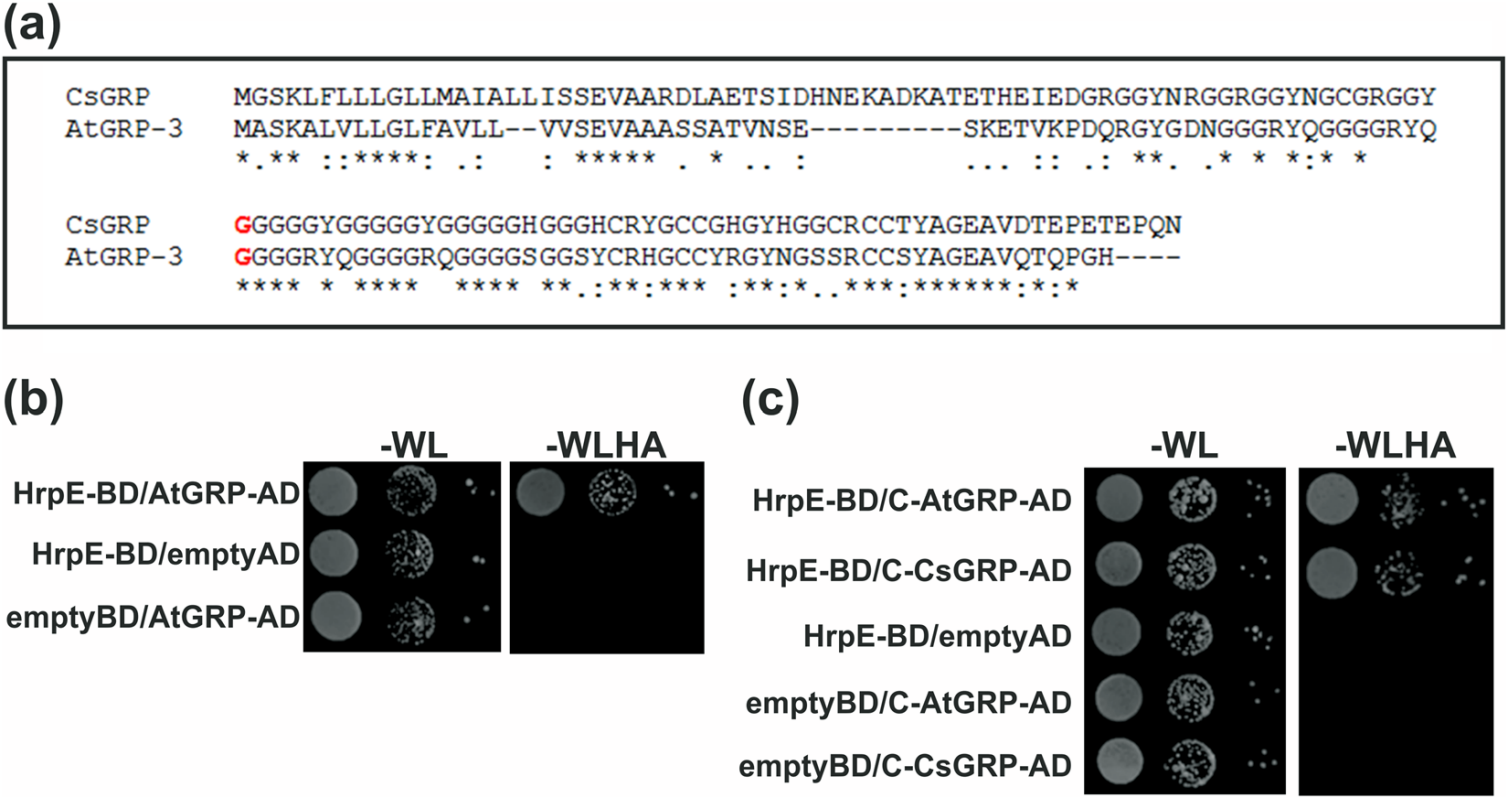
Yeast Two Hybrid assays of the interactions HrpE-AtGRP-3 and HrpE with the C-terminal end of CsGRP and AtGRP-3. (**a**) The sequences of CsGRP and AtGRP-3 were aligned using ClustalW program. Asterisks (*) indicate identical amino acids, colons (:) are conservative replacements, full stops (.) are semi-conservative replacements. In red are shown the Gly72 of CsGRP and the Gly60 of AtGRP-3 where the highly conserved C-terminal ends start. (**b**) Representative photograph of YTH assay showing that HrpE interacts with AtGRP-3. Yeast cells were co-transformed with: HrpE-BD/AtGRP-3-AD, HrpE-BD/empty-AD and empty-BD/AtGRP-3-AD. Yeast growth (serial 1:10 dilutions) is shown in −WL plates and in −WLHA plates containing 35 mM 3AT. (**c**) Representative photograph of YTH assay showing that HrpE interacts with the conserved C-terminal ends of CsGRP and AtGRP-3 (C-CsGRP and C-AtGRP-3, respectively). Yeast cells were co-transformed with: HrpE-BD/C-AtGRP-3-AD, HrpE-BD/C-CsGRP-AD, HrpE-BD/empty-AD, empty-BD/AtGRP-3-AD and empty-BD/C-CsGRP-AD and serial 1:10 dilutions are shown in −WL plates and in −WLHA plates containing 35 mM 3AT.

*AtGRP-3* gene from *A*. *thaliana* (At2g05520), encodes a protein which is the closest homologue to CsGRP (76% of similarity and 48% of identity). Especially, the C-terminal region of CsGRP (from Gly72) and AtGRP-3 (from Gly60) are highly conserved (85% of similarity and 65% of identity) (Fig. 5a). A positive interaction of AtGRP-3 with HrpE (HrpE-BD/AtGRP-AD) was also observed by YTH (Fig. 5b). In addition, the interactions of HrpE with both highly conserved C-terminal regions of AtGRP-3 (HrpE-BD/C-AtGRP-AD) and CsGRP (HrpE-BD/C-CsGRP-AD) were tested by YTH assays and positive interactions were observed (Fig. 5c).

**Figure 6:**
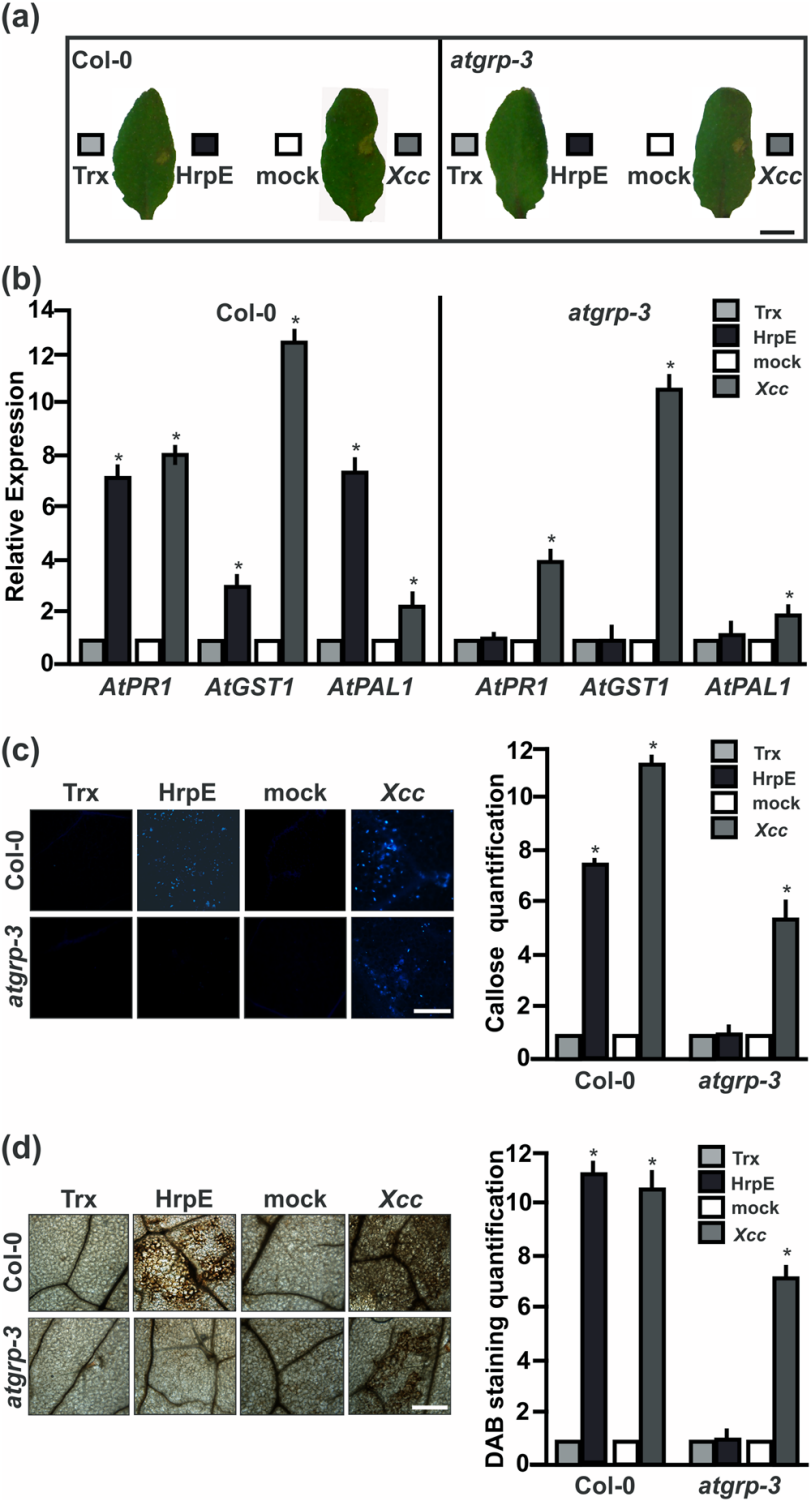
AtGRP-3 is involved in plant defense responses elicited by HrpE. (**a**) Representative photographs of leaf responses in *A*. *thaliana* Col-0 and *atgrp-3* to the infiltration of pure 2.5 µM HrpE or Trx (control) and *Xcc* (10^7^ cfu/mL) or 15 mM NaCl as control (mock) 1 dpi. Bar represents 1 cm. (**b**) Analysis qRT-PCR of the expression levels of genes related with defense responses in Arabidopsis leaves, infiltrated as in (**a**). RNA was extracted from leaves 4 hpi. Darker bars indicate the expression levels of the genes after HrpE or *Xcc* infiltration relative to the expression levels of the Trx or NaCl controls (lighter bars), respectively. Values are the means of four biological replicates with three technical replicates each. Error bars indicate standard deviations. Results were analyzed by one-way ANOVA (*p* < *0*.*05*). (**c**) Representative fluorescence microscopy photographs of aniline blue staining of callose deposition in *A*. *thaliana* Col-0 or *atgrp-3* leaves infiltrated with 2.5 µM HrpE, Trx (control), *Xcc* at 10^7^ cfu/mL or 15 mM NaCl (mock) 8 hpi (Bar indicates 20 μm). The right panels show the quantification of callose intensities of HrpE and *Xcc* (darker bars) relative to controls (lighter bars). (**d**) Representative photographs of DAB stained leaves infiltrated as in (**c**) (Bar indicates 20 μm). In *A*. *thaliana*, H_2_O_2_ production is observed as brown precipitates in leaf tissues. The right panels show the quantification of DAB staining of HrpE (darker bars) relative to controls (lighter bars). For both callose and DAB intensities quantifications, the means were calculated from 25 photographs obtained from different treated leaves from three independent experiments. Error bars indicate standard deviations. Asterisks indicate significant differences based on one-way ANOVA (*p* < *0*.*05*).

These results indicate that AtGRP-3 can also recognize HrpE, therefore, defense responses to HrpE were analyzed in *A*. *thaliana* wild type Col-0 and in a single T-DNA homozygous knockout line in the *AtGRP-3* gene (*atgrp-3*). qRT-PCR assays corroborated that this *atgrp-3* mutant line lacks *AtGRP-3* expression and therefore represents a null mutant (Fig. S4). *A*. *thaliana* Col-0 plants infiltrated with HrpE showed a noticeable response but, no response to this protein was observed in leaves of the *atgrp-3* mutant (Fig. 6a). Both *A*. *thaliana* cultivars responded to the infiltration with a *Xcc* suspension showing an HR response (Fig. 6a). The expression of *A*. *thaliana* defense-related genes^11^, such as *PR1*, *PAL1* and *GST1* was analyzed in these plants by qRT-PCR. HrpE treatment induced the expression of these genes in Col-0 plants (*p* < *0*.*05)*, but not in the *atgrp-3* knockout line (Fig. 6b). In *Xcc*-infiltrated plants, the expression of defense genes was increased in both lines (*p* < *0*.*05)* (Fig. 6b). HrpE treatment did not induce the production of callose depositions and H_2_O_2_ in the *atgrp-3* mutant compared to Col-0 line and Trx-infiltrated controls (Fig. 6c,d). The *atgrp-3* mutant line responded to *Xcc* treatment but showed lower levels of callose deposition and H_2_O_2_ than the Col-0 line (*p* < *0*.*05)* (Fig. 6c,d).

### Contribution of HrpE N-terminal and C-terminal regions to the plant defense response

**Figure 7:**
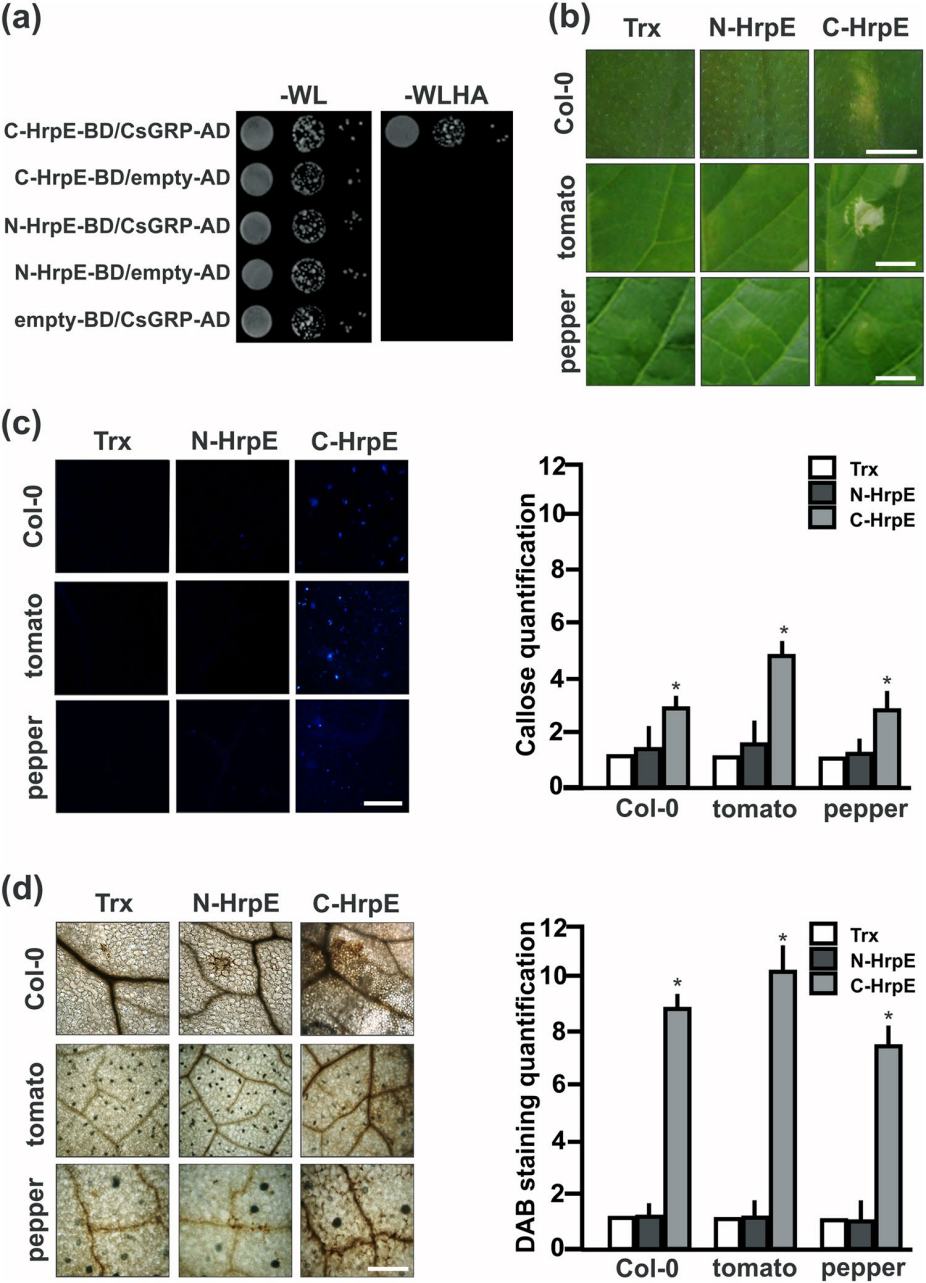
Analysis of HrpE N- and C-terminal regions in the defense response. (**a**) Yeast two-hybrid assays of HrpE N-terminal region (N-HrpE-BD) and HrpE C-terminal region (C-HrpE-BD) against CsGRP-AD. Serial dilutions (1:10) of co-transformed yeast were plated on −WL and on −WLHA plates containing 35 mM AT. Co-expression of N-HrpE-BD/empty-AD, C-HrpE-BD/empty-AD and empty-BD/CsGRP-AD were used as controls. (**b**) Representative photographs of *A*. *thaliana*, tomato and pepper plant responses to infiltrations with 2.5 µM N-HrpE, C-HrpE and Trx (control) 16 hpi. Bar indicates 0.5 cm. (**c**) Representative photographs of callose staining of leaves infiltrated as in (**b**). Bar indicates 20 μm. Right panel shows the quantification of callose deposits in infiltrated Col-0, tomato, and pepper tissues. Asterisks indicate significant differences based on one-way ANOVA (*p* < *0*.*05*). (**d**) Representative photographs of DAB stained leaves, infiltrated as in (**b**). Bar indicates 20 μm. Right panel shows the quantification of DAB production is these leaves. Asterisks indicate significant differences based on one-way ANOVA (*p* < *0*.*05*). For both, callose and DAB intensities quantifications, the means were calculated from 25 photographs obtained from different treated leaves from three independent experiments. Error bars indicate standard deviations.

HrpE protein is highly conserved among many Xanthomonas species, especially the C-terminal region (^8^ and Fig. S5a). An homology structural model of *Xcc* HrpE obtained using the Robetta server suggests that HrpE is an α-helix rich protein (Fig. S5b). Based in this model, HrpE was dissected into an N-terminal region (residues 1–52) and the conserved C-terminal region (residues 53–93), without disrupting any α-helical structure. YTH assays were performed to determine whether these regions interact with CsGRP (Fig. 7a). A positive interaction was observed only between the C-terminal region of HrpE and CsGRP (C-HrpE-BD/CsGRP-AD) (Fig. 7a). Furthermore, the N- and C-terminus portions of HrpE were expressed as Trx-^6^His fusions in *E*. *coli* and purified. Leaves of citrus, *A*. *thaliana*, tomato and pepper were infiltrated with the recombinant proteins. Only the C-terminal region of HrpE caused visible lesions in non-host plants (Fig. 7b). On the other hand, the N-terminal region of HrpE did not elicit a significant response in any plant assayed (Fig. 7b). Aniline Blue (Fig. 7c) and DAB staining (Fig. 7d) revealed that the C-terminal HrpE induced greater callose deposition and production of H_2_O_2_ in leaves of non-host plants than the control (*p* < *0*.*05*). Leaves infiltrated with N-terminal HrpE gave results very similar to those observed for the Trx control.

## Discussion

Xanthomonas bacteria infect host plants through the secretion of effector proteins by the T3SS. In the citrus canker pathogen *Xcc*, a major role for the T3SS in pathogenicity and HR^5^ and modulation of bacterial biofilm formation^6^ have been previously demonstrated. T3SS HrpE protein is present and conserved in several Xanthomonas species. In *Xcv*, the HrpE protein was identified as the main structural component of the Hrp pilus and its role in the secretion of effector proteins has been well established^8^. Here, whether plants can recognize this pilus protein to elicit a defense response against Xanthomonas pathogens was investigated. The results showed that HrpE protein elicits defense responses in both citrus and non-host plants. This was evidenced upon HrpE infiltration in different plants in the form of the development of visible lesions and defense response markers such as the accumulation of callose deposits and enhanced H_2_O_2_ production. Moreover, infiltration of HrpE by itself was sufficient to induce expression of numerous citrus and non-host plant defense-related genes. It is noteworthy, that defense responses in citrus were weaker than in non-host plants. This can be attributed to the fact that citrus leaf is naturally more resistant to elicitors of defense responses than non-host plants^11,12^. Supporting a role for HrpE as an enhancer of defense responses, pre-infiltration of citrus and tomato leaves with HrpE impaired the virulence of *Xcc* and *Xcv* on these plants, respectively. This feature is novel for Hrp pilus proteins, being even more remarkable considering that the effect can be observed in different plants.

Several surface bacterial molecules that are needed for the interaction with eukaryotic hosts are important inducers of plant and animal innate immunity. These molecules increase the plant defense response by interacting with host cell surface receptor proteins which are able to trigger the signaling that ends in the immune response^10^. Regarding bacterial proteins belonging to the T3SS needle, a recent report shows that the needle tip protein IpaD of *Shigella flexneri*, causative agent of bacillary dysentery, can trigger lymphocytes B cell death but in this case, the presence of bacterial co-signals is required to sensitize these host cells to apoptosis^19^. Therefore, to our knowledge this study is the first report in which an injectisome protein such as HrpE elicits the plant immune response, warning the plant of bacterial presence.

The search for plant molecules that may interact with HrpE led to the detection of CsGRP. This result was revealed by YTH and confirmed by Far-Western and *in planta* BiFC assays. The GRP superfamily is characterized by the presence of variable semi-repetitive glycine-rich motifs. Based on these variations, this superfamily has been further divided into five distinct classes (I-V)^20^ and has several functions in plants including plant defense against abiotic and biotic stress^21^. The CsGRP closest homologue in *A*. *thaliana* is AtGRP-3 and the two proteins displayed a high sequence identity, mainly at the C-terminal end. AtGRP-3 belongs to the Class II GRPs and is strongly expressed in leaves and stems^22^. The protein sequence contains a putative signal peptide that predicts an apoplastic localization followed by a glycine-rich region with a GGXXXGG motif and a cysteine-rich C-terminus. AtGRP-3 has been shown to localize mainly in the apoplast and cell wall and, in a small part, on the plasma membrane^23^. CsGRP is also predicted to localize at the apoplast by PSORT (www.psort.org), the place where it may interact with the bacterial HrpE and enhance plant defense responses. In this work, HrpE-CsGRP interaction *in planta* was confirmed by BiFC localizing at the apoplast and this result suggests that CsGRP localization occurs outside the plant cell similar to AtGRP-3. Even if HrpE does not contain a plant secretion signal, it may be put forward that the bacterial secretion signals present in HrpE^24^ are recognized by the plant secretion machinery as it has been observed for a bacterial toxin secreted in maize^25^. Also, it has been reported that plant proteins overexpression can cause protein miss-sorting to earlier or even more distal compartments in the trafficking system, and this may also be taking place^26^, even if these suggestions have to be further analyzed.

Moreover, HrpE interacted with AtGRP-3, and the highly conserved C-terminal regions of both proteins CsGRP and AtGRP-3 were sufficient for these interactions. After HrpE infiltration, *A*. *thaliana atgrp-3* mutant plants did not show visible lesions, callose deposits or H_2_O_2_ production, and genes involved in the defense response were not modified. The *atgrp-3* mutant showed a weaker defense response to *Xcc* than wild type plants; these results suggest that other PAMPs in *Xcc* can still trigger defense response in this mutant, which is mediated by a defense signaling parallel to HrpE-AtGRP-3 triggered signaling. These results indicate that AtGRP-3 is involved in the mechanism that enhances Arabidopsis defense response against HrpE.

In Arabidopsis it was demonstrated that AtGRP-3 has a negative role decreasing the plant defense responses. This negative role is attributed to the fact that the C-terminal end of AtGRP-3 binds to a cell wall associated kinase AtWAK1, which is a positive regulator of these responses, and this interaction makes AtWAK1 unable to enhance plant defense responses^23^. In this context, we propose that the interaction of HrpE with AtGRP-3 at the C-terminal end of this protein may change the affinity of AtGRP-3 for other proteins, such as the interaction described with AtWAK1, culminating in a enhancement of plant defense response. This hypothesis and whether CsGRP/AtGRP-3 homologues from different plants may interact with HrpE will be the focus of further studies.

In *Xcv*, the secretion of HrpE depends on a T3SS secretion signal at its N-terminus^27^. Intriguingly, comparison of the HrpE sequences from several Xanthomonas species showed a variable N-terminal half (residues 1–52), in contrast to the more highly conserved C-terminal half (residues 53–94)^8^. Structural predictions showed that HrpE is predominantly α-helical with the highest helical content at the conserved C-terminus. The ability of these N- and C- terminal regions of HrpE to interact with CsGRP and to elicit a defense response in plants was analyzed. Results showed that C-terminal domain, but not the N-terminal region, can interact with CsGRP. In addition, leaves of non-host plants infiltrated with the C-terminal region showed visible lesions, callose deposition and H_2_O_2_ production. To notice is that visible responses and callose deposition to C-HrpE infiltration were lower than the ones elicited by the complete protein, even if DAB staining remained similar in both treatments. Therefore, even if the complete protein is required for the full reaction, C-terminal region is able to cause similar hydrogen peroxide production as a major defense response. These results suggest that HrpE from several Xanthomonas may elicit plant defense responses through the conserved C-terminal region.

This is the first report of the participation of a pilus protein, such as HrpE, as an elicitor of plant defense responses. Overall, the results of this work suggest that plants have developed the ability to recognize the conserved C-terminal part of HrpE as a danger signal to defend themselves against Xanthomonas species and that plant GRPs may mediate this defense response. Based on these results, HrpE arises as a key pathogen protein, which can be recognized by several plants and trigger defense response induction. Therefore, the transgenic expression of this protein could be tested as a novel strategy for obtaining resistant plants.

## Methods

### Strains, culture conditions and media

*Xcc* strain *Xcc*99-1330, isolated from *Citrus sinensis* (INTA Bella Vista, Argentina) the derivatives *Xcc*HrpG^+^ and *Xcc*HrpG^-^ strains^16^ and the *Xcv* Bv5-4a strain (INTA Bella Vista, Argentina) were grown at 28 °C in Sucrose Broth (SB)^28^. Antibiotics were used at the following final concentrations: ampicillin, 25 µg/mL; kanamycin, 40 µg/mL; gentamycin, 20 µg/mL and chloramphenicol, 30 µg/mL. Agrobacterium tumefaciens strain GV3101 was cultured at 28 °C in LB medium using: rifampicin, 100 µg/mL and gentamycin, 20 µg/mL. A. tumefaciens bearing AvrXacE2-GFP was grown using rifampicin, 100 µg/mL and gentamycin, 20 µg/mL and spectinomycin 100 µg/mL^18^. A. tumefaciens strain C58C1, containing the silencing suppressor p19 under the control of the 35 S promoter, was cultured in LB medium with tetracycline 5 µg/mL and kanamycin 50 µg/mL.

### Expression and purification of recombinant proteins

The full-length *hrpE*, *NhrpE* and C*hrp*E genes were amplified by PCR from *Xcc* genomic DNA using the oligonucleotide combinations HrpEf-HrpEr; HrpEf-NHrpEr and CHrpEf-HrpEr, respectively (Table S2). All amplicons were cloned into pET32a vector (Novagen) previously digested with the restriction enzymes *BamH*I and *Hind*III. Full-length cDNA of *CsGRP* was amplified from *C*. *sinensis* cDNA obtained from *Xcc*-infected leaves tissues with the oligonucleotides CsGRPf and CsGRPr (Table S2). The PCR product was cloned in the pGEX-4T3 vector (GE Healthcare) digested with *Sal*I and *Not*I. All the constructs were transformed into *Escherichia coli* strain BL21 (pLysS), and the synthesis of recombinant proteins was induced by 0.5 mM isopropyl-β-D-1-thiogalactopyranoside (IPTG) for 16 h at 18 °C. The proteins were purified by affinity chromatography from the soluble fraction of the bacterial lysate. HrpE-Trx-^6^His (HrpE), NHrpE-Trx-^6^His (N-HrpE), CHrpE-Trx-^6^His (C-HrpE) and Trx-^6^His (Trx) were purified with a Ni^2+^-nitrilotriacetate (Ni-NTA) agarose column (Qiagen) and CsGRP-GST (CsGRP) and GST with a Glutathione Sepharose column (GE Healthcare). The proteins were dialyzed for 24 h with PBS buffer.

### Structural modeling of *Xcc* HrpE

The modeling of HrpE structure was performed with the Full-chain Structure Prediction Server Robetta (available at http://robetta.bakerlab.org).

### Plant material and protein plant inoculations

*Citrus sinensis* cv. Valencia (citrus) were grown in a green house at 26 ± 2 °C and *Solanum lycopersicum* cv. Victoria (tomato), *Capsicum annuum* cv. Grossum (pepper) and *Nicotiana benthamiana* at 24 ± 2 °C, all of them with a photoperiod of 16 h. Seeds of *Arabidopsis thaliana* cultivars Col-0 and knockout line *atgrp-3* (SALK_012941C) available in the *Arabidopsis Biological Resource Center*, were sown in soil in 10-cm pots and grown in a controlled environment chamber at 23 ± 2 °C with a photoperiod of 16 h. Proteins were infiltrated with needleless syringes at the indicated concentrations.

### Callose staining

This assay is based on the staining of callose with aniline blue and cytological observations at the sites of infiltration by UV fluorescence microscopy. Citrus, tomato, pepper and *A*. *thaliana* leaves were infiltrated with 2.5 µM of HrpE, N-HrpE, C-HrpE and Trx as control, and with 10^7^ cfu/mL *Xcc* and 15 mM NaCl as control, and at 8 and 16 hpi callose staining was performed as described previously^11^. The stained leaves were examined and photographed by UV fluorescence microscopy. Callose intensity was calculated from digital photographs by the number of blue pixels relative to the total number of pixels covering the plant material, using Photoshop CS3 software^11^. The results shown are related to the control treatment in which the callose intensity was considered to be one. Average callose measurements were based on at least 25 photographs from different treated leaves from three independent experiments.

### 3,3′-Diaminobenzidine (DAB) staining

To visualize H_2_O_2_ accumulation, citrus, tomato, pepper and *A*. *thaliana* leaves were infiltrated with with 2.5 µM of HrpE, N-HrpE, C-HrpE and Trx and with 10^7^ cfu/mL *Xcc* and 15 mM NaCl and, after 8–16 hpi were stained with DAB (Sigma, St Louis, USA) as described previously^13^. The stained leaves were observed and photographed in an optical microscope. DAB intensity was calculated from the digital photographs by the number of brown pixels as described previously^13^. Average DAB measurements were calculated from at least 25 photographs from different treated leaves from three independent experiments.

### RNA preparation and quantitative reverse transcription-PCR (qRT-PCR)

Total RNA from treated leaves were isolated using TRIzol® reagent (Invitrogen), according to the manufacturer’s instructions. qRT-PCRs were performed as described previously^11^, with the specific oligonucleotides detailed in Table S2. Values are the means of four biological replicates with three technical replicates each.

### Analysis of *Xcc* and *Xcv* growth in citrus and tomato leaves pre-treated with HrpE

Ten citrus leaves were infiltrated or tomato leaves sprayed with 1 µM HrpE and Trx, 15 mM NaCl and 10^7^ cfu/mL suspensions of the *Xcc*hrpB^−^ strain as controls^13^. Tomato leaves were sprayed instead of infiltrated since these leaves are more fragile than citrus leaves and their tissues do not support the infiltration with HrpE, Trx or *Xcc*hrpB^−^ and the subsequent infiltration with Xcv. After 16 hpi, these leaves were infiltrated with 10^6^ cfu/mL *Xcc* or *Xcv* suspension with needleless syringes. Growth assays were performed at different times post-infection by grinding 0.8 cm diameter leaf discs in 1 mL of 15 mM NaCl, followed by serial dilutions and plating onto SB agar plates. Colonies were counted after 48 hpi of incubation at 28 °C, and the results are presented as log cfu/cm^2^ of leaf tissue.

### Yeast two-hybrid assays

The full-length *hrpE*, *NhrpE* and *ChrpE* genes were amplified by PCR from *Xcc* genomic DNA using the oligonucleotide combinations HrpE-BDf/HrpE-BDr; HrpE-BDf/N-HrpE-BDr and C-HrpE-BDf/HrpE-BDr, respectively (Table S2). All amplicons were cloned into pOBD vector^29^, previously digested with the restriction enzymes *EcoR*I and *Pst*I and transformed into the *Saccharomyces cerevisiae* strain PJ694a^30^. The strains were grown in the yeast medium YAPD or synthetic complete (SC) medium as described previously^31^. When indicated, SC medium was prepared lacking one or more specific components: Tryptophan (−W), Leucine (−L), Histidine (−H) and Adenine (−A). In the case of growth on solid medium, 1.6% Bacto Agar and 3AT (see below) were added. To detect *C*. *sinensis* proteins that interact with HrpE, a *C*. *sinensis* cDNA library containing approximately 0.8 × 10^6^ independent clones in the pOAD vector was used. This library was constructed and kindly donated by Raúl Andrés Cernadas, Cássia Docena and Celso Eduardo Benedetti at the Laboratório Nacional de Luz Síncrotron (Campinas, Brazil), and has been described elsewhere^32^. The pOBD-HrpE plasmids was transformed in the PJ694a strain by using the PEG3350–lithium acetate protocol^33^ and selected on SC–W plates at 30 °C for 2–4 days. These cells were then employed in high efficiency transformations with the pOAD *C*. *sinensis* cDNA library using 30 μg of plasmid DNA and the 30× scale-up procedure^33^. To determine the amount of 3AT (as inhibitor of the autoactivation of the system) to be used for each bait, yeast cells transformed with the pOBD-bait plasmid were plated onto SC–WH medium containing 3, 5, 35, 55 and 100 mM 3AT and incubated for 5 days at 30 °C. pOAD plasmids recovered from positive clones were sequenced using a pOAD-specific primer. Sequences were analyzed by comparison with the available *C*. *sinensis* expressed sequence tag (EST) database. The genes that codify for CsGRP, C-CsGRP, AtGRP-3 and C-AtGRP-3 were cloned in the pOAD vector using the oligonucleotide combinations CsGRP-ADf/CsGRP-AD, C-CsGRP-ADf/CsGRP-ADr, AtGRP-3ADf/AtGRP-3ADr and C-AtGRP-3ADf/AtGRP-3ADr, respectively (Table S2). Direct interaction of proteins was investigated by co-transformation of the respective plasmids in the PJ694a yeast strain, followed by selection of transformants on SC-WL at 30 °C for 3 days and subsequent transfer to medium lacking W, L, H and A (supplemented with 35 mM 3AT) for growth selection and *His* and *Ade* activity testing of interacting clones. Controls are detailed in the text, briefly they include the co-transformation in PJ694a cells with the different construct in the pOAD plus the empty-BD or the different construct in the pOBD plus the empty-AD and the growth on the selective media as detailed above.

### Polyclonal antibodies production

The purified HrpE and GST were used to immunize New Zealand rabbits to obtain polyclonal antibodies as described previously^34^.

### Preparation of Hrp-pilus and Far Western assays

Hrp-pilus preparations were performed from *Xcc*hrpG^+^ cells grown statically in XVM2 medium for 7 days at 30 °C with a deoxycholate-sucrose density gradient, as described previously^35^. As control, similar preparations were performed with *Xcc*hrpG^−^ grown in XVM2 medium and with *Xcc*hrpG^+^ grown in rich SB medium. Purified fractions were resolved by 15% Tricine SDS-PAGE, transferred to nitrocellulose membranes under native conditions using 24 mM Tris and 194 mM glycine buffer and revealed with anti-HrpE rabbit polyclonal antibody. For Far Western assays the membranes were blocked with PBSt-milk (137 mM NaCl, 2.7 mM KCl, 10 mM Na_2_HPO_4_.2H_2_O, 2 mM KH_2_PO_4_ pH 7.4, supplemented with 0.05% Tween-20 and 5% powdered milk) for 1 h and then probed with 50 μg mL^−1^ of CsGRP-GST and GST in 50 mM Tris base pH 7, 25 mM KCl, 1 mM DTT, 0.1% Tween-20 and 2% powdered milk buffer for 16 h at 4 °C. Membranes were then washed four times with PBSt buffer and incubated with anti-GST for 1 h, then washed and incubated with anti-rabbit 1:3000. Alkaline phosphatase activity was assayed using NBT/BCIP system (Sigma). Experiments were repeated three times with similar results.

### *In planta* BiFC Assays

The full-length *hrpE* and *CsGrp* genes were amplified by PCR from *Xcc* genomic DNA and citrus cDNA using the oligonucleotide combinations HrpEBiFCf-HrpEBiFCr; CsGrpBiFCf-CsGrpBiFCr, respectively (Table S2). HrpE was cloned fused to the non-fluorescent N-terminal fragment of venus fluorescent protein (nVenus) (HrpE-nVenus) and CsGRP was fused to the non-fluorescent C-terminal fragment of CFP (cCFP) (CsGRP-cCFP) into commercial pSAT vectors^36^. In order to express proteins in plants, digested products were cloned in the pCHF3 binary vector^37^ using *Sac*I and *XbaI* enzymes. Plasmid vectors pCHF3-HrpE-nVenus, pCHF3-CsGRP-cCFP, pCHF3-nVenus or pCHF3-cCFP were electroporated into *Agrobacterium tumefaciens* strain GV3101 with a Gene Pulser II (Bio-Rad,Hercules, CA, USA) according to the manufacturer’s instructions. For infiltration assays, *A*. *tumefaciens* strains were grown in LB broth medium supplemented with the appropriate antibiotics, at 28 °C for 18 h, diluted 1:100 and incubated at 28 °C to OD600 nm of 1.0. The resulting cultures were harvested by centrifugation and resuspended in sterile buffer [10 mM MgCl_2_and 100 mM acetosyringone]. After 3 h incubation at 30 °C, cultures were co-infiltrated at a 1:2 ratio with Agrobacterium carrying the silencing suppressor p19. Cells were used to infiltrate leaves of 4-5-week-old *N*. *benthamiana* using needleless syringes. When indicated, plasmolysis was performed by treatment with 0.8 M mannitol for 20 min. Fluorescence by Venus in plant cells was assessed at 72 h post-infiltration. Briefly, leaf sections were excised and mounted in glycerol 10% (v/v) for observation under a Nikon Eclipse TE-2000-E2 (Nikon Instruments Inc., Melville, NY, USA) confocal laser scanning microscope. Pairing the cCFP (from amino acids 155–238) with nVenus (from amino acids 1–173) results in yellow fluorescence. Excitation of the fluorophore was done at 488 nm using an argon laser. Emission was captured with a 505–530 nm pass filter. GFP was excited with at 488 nm and the emission filter wavelengths were 497–526 nm.

### Statistical analysis

In all figures, bars are the mean of the data and error bars are the standard deviation and asterisks (*) indicate significant difference *p* < *0*.*05* analyzed by one-way analysis of variance (ANOVA).

### Availability of Data and Materials

All data generated or analyzed during this study are included in this manuscript and it supplementary information files, or is available upon request.

## Electronic supplementary material

Supplementary Information
