# Supplementary material for: HrpE, the major component of the Xanthomonas type three protein secretion pilus, elicits plant immunity responses
Source: Sci Rep. 2018 Jun 29;8:9842. doi: 10.1038/s41598-018-27869-1 (PMC6026121; doi:10.1038/s41598-018-27869-1)
Supplement: Supplementary file 1 — Supplementary Information [file 41598_2018_27869_MOESM1_ESM.pdf]

Supplementary Information

HrpE, the major component of the Xanthomonas type three protein secretion pilus, elicits plant immunity responses

Natalia Gottig[¶]*, Cecilia V. Vranych[¶], Germán G. Sgro, Ainelén Piazza, Jorgelina Ottado*

Instituto de Biología Molecular y Celular de Rosario, Consejo Nacional de Investigaciones Científicas y Técnicas (IBR-CONICET) and Facultad de Ciencias Bioquímicas y Farmacéuticas, Universidad Nacional de Rosario (UNR). Ocampo y Esmeralda, Rosario, Argentina.

* Corresponding authors: Dra. Natalia Gottig (gottig@ibr-conicet.gov.ar) and Dra. Jorgelina Ottado (ottado@ibr-conicet.gov.ar)

[¶] These authors contributed equally to this work

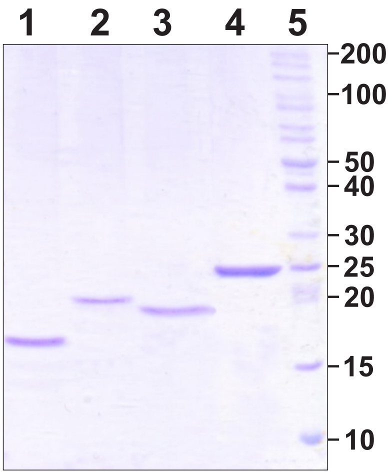

Figure S1. SDS-PAGE analysis of purified proteins. Purity and protein molecular weight were visualised by SDS-PAGE in a 15% polyacrylamide gel. 20 μl of 5 μM protein solutions were loaded in each lane. Lane 1: Trx, lane 2: N-HrpE, lane 3: C-HrpE, lane 4: HrpE and lane 5: marker (PageRuler. Thermo Scientific).

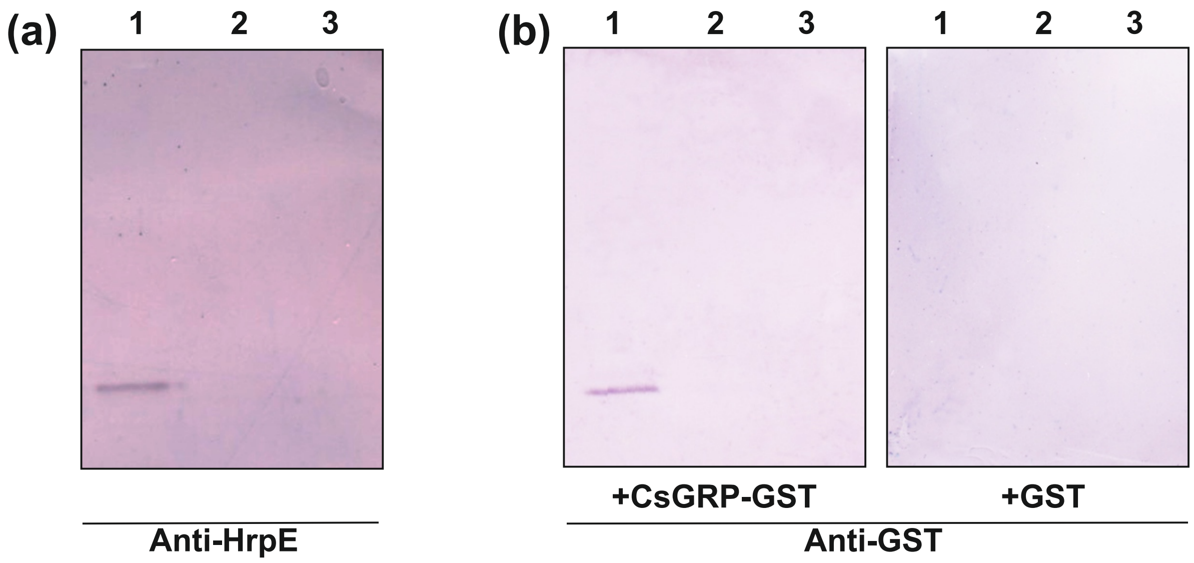

Figure S2. Full-length Western and Far Western Blots as in Figure 4 (b) and (c). (**a**) Purification of the Hrp-pilus from *Xcc*hrpG^{+} (lane 1) and *Xcc*hrpG^{-} (lane 2) grown in XVM2 medium and from *Xcc*hrpG^{+} (lane 3) grown in SB. Proteins obtained from pilus preparations were analyzed by Tricine-SDS-PAGE and Western blot revealed with anti-HrpE polyclonal antibody. (**b**) Far Western blots showing interactions between HrpE present in the Hrp-pilus preparations and CsGRP-GST. Nitrocellulose membranes similar to that showed in (**b**) were incubated with 50 µg CsGRP-GST or GST as a control and, after washing, probed with anti-GST polyclonal antibody.

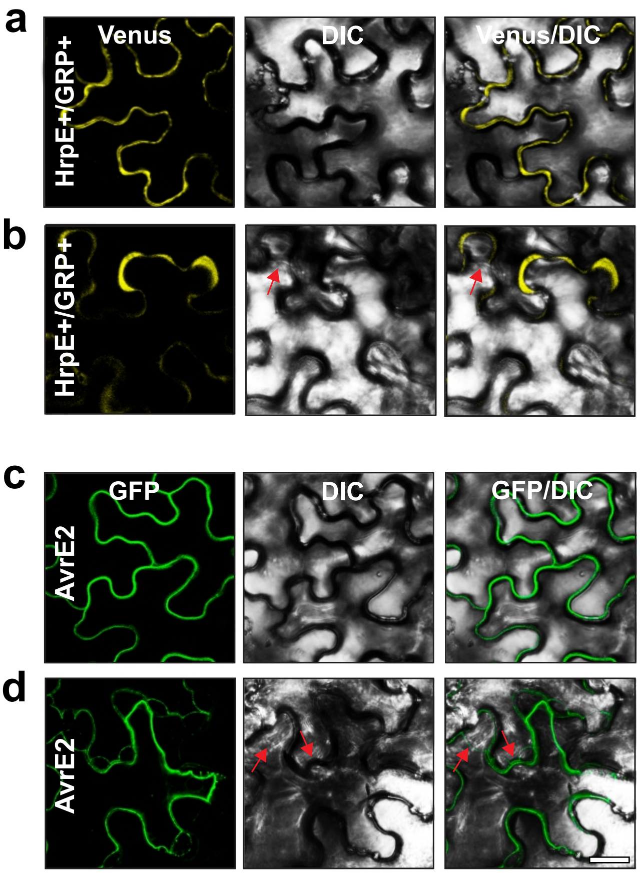

Figure S3. Localization of HrpE/CsGRP interaction upon plasmolysis. (**a**) Localization of HrpE+/CsGRP+ interaction without plasmolysis and (**b**) upon plasmolysis induced by 0.8 M mM mannitol for 20 min. The retracted plasma membranes are shown by red arrows and they do not have associated fluorescence (**b**). (**c**) Localization of the membrane protein, AvrXacE2-GFP (AvrXacE2). (**d**) Upon plasmolysis; fluorescence associated with the retracted plasma membrane from the cell wall is shown by red arrows. Analyses were performed by Confocal laser-scanning microscopy Scale bars represent 25 μm. DIC: Differential Interference Contrast.

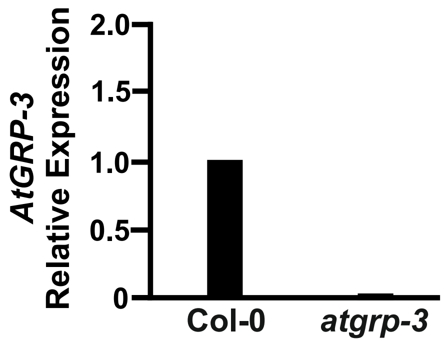

Figure S4. Analysis of *AtGRP-3* expression in the Arabidopsis null mutant *atgrp-3*. RNA was extracted from leaves of *A. thaliana* Col-0 and *atgrp-3* mutant line and qRT-PCR assays were performed. Expression in Col-0 was considered as 1. Values are the means of three biological replicates with three technical replicates each.

a

```
Xcv     MQIFPEVSSWRSRVGQGMDCFTGGLSNGISGAAALSGANGQMDSLLGDMSAS
Xcc     MELLPQISSIKSRFDQGTDAYTGGVSGGISGEAALTGANGQMSSLISDMNAS
Xam     MELFPQMSSLKSRFDQGTDAYTGGVSGGISGASALSGANGQMSSLISDMTAS
Xcvi    MELFPQMSSLKSRFDQGTDAYTGGVSGGISGTSALSGANGQMSSLISDMTAS
Xcb     MEIFPQISSIKSRFGQGIDGYTGGVSGGISGEAALYGSNRQMSSLISDLTAS
Xoo     MEILPQISSLNSRFEQGMDGYTGGVAGGISGASALSGSNGQMGSLLSDMGAS
Xcm     MELFPQISSIRSRFVQGMDGYTGGVSGGISGASALSGSNGQMSSLISDLTAS
Xaa     MELFPQISSLNSRFEQGMDGYTGGVSGGISGAGALSGANGQMNSLISDMAAS
Xac     MELFPQISSLNSRFEQGMDGYTGGVSGGISGAAALSGADGQMSSLISDMTAS
Xag     MELFPQISSLNSRFEQGMDGYTGGVSGGISGADALSGANGQMSSLISDMTAS
        *:::*::** .**. *  * :***::.****  ** *:: **.**:.*: **

Xcv     DEAQKSMNNKITMLKNDLDFNVALNKFIGKAGDNAKQLVGQ
Xcc     DEAQKSMNNKITQLKNDLDFNVALNKFIGKAGDNAKQLVGQ
Xam     DEAQKSMNNKITQLKNDLDFNVALNKFIGKAGDNAKQLVGQ
Xcvi    DEAQKSMNNKITMLKNDLDFNVALNKFIGKAGDNAKQLVGQ
Xcb     DEAQKSLNNKITMLKNDLDFNVVLNKFIGKAGDNAKQLVGQ
Xoo     DEAQKSMNNKITQLKNDLDFNVALNKFIGKAGDNAKQLVGQ
Xcm     DEAQKSMNNKITMLKNDLDFNVALNKFIDKAGDNAKQLVGQ
Xaa     DEAQKSMNNKNTMLKNDLDFNVALNKFIGKAGDNAKQLVGQ
Xac     DEAQKSLNNKITMLKNDLDFNVALNKFIGKAGDNAKQLVGQ
Xag     DEAQKSMNNKITMLKNDLDFNVALNKFIGKAGDNAKQLVGQ
        ******:*** * *********.*****.************
```

b

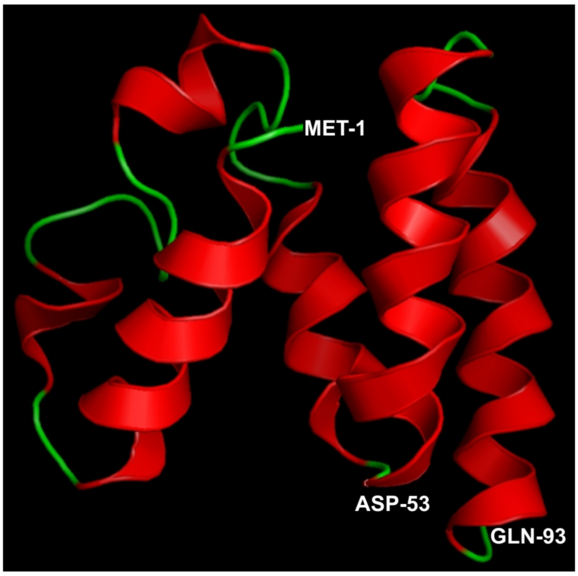

Figure S5. Conservation and structural modeling of *Xcc* HrpE. (**a**) The sequences of HrpE proteins present in different Xanthomonas were aligned using ClustalW program. Asterisks (*) indicate identical amino acids, colons (:) are conservative replacements, full stops (.) are semi-conservative replacements. In red is shown the Asp53 amino acid. Xcv: *Xanthomonas campestris* pv. *vesicatoria*, Xcc: *Xanthomonas citri* subsp*. citri*, Xam: *Xanthomonas axonopodis* pv*. malvacearum*, Xcvi: *Xanthomonas campestris* pv*. viticola*, Xcb: *Xanthomonas campestris* pv*. begonia*, Xoo: *Xanthomonas oryzae* pv*. oryzicola*, Xcm: *Xanthomonas citri* subsp*. malvacearum*, Xaa: *Xanthomonas alfalfae* subsp*. alfalfa*, Xac: *Xanthomonas axonopodis* pv*. citrumelo*, Xag: *Xanthomonas axonopodis* pv*. glycines*. (**b**) Schematic representation of the structural model of HrpE created with the Robetta server. The protein is predicted to fold in several α-helical regions but with more disordered portions at the N-terminal region. The initial methionine (MET-1), the 53 aspartic acid where the protein was dissected (ASP-53) and the final glutamine (GLN-93) are labeled in white.

Table S1. Identified *Citrus sinensis* proteins that interact with HrpE by yeast two-hybrid assay.

**Predicted protein**	**Accession number**	**Initial codon**
Glycine-rich protein-like	XP_006469561.1	1
Small heat shock protein	XP_006473921.1	1
Putative metallothionein-like protein	ABL67648.1	1
Probable receptor-like protein kinase At5g24010	XP_024044862.1	2
Protein YLS9-like	XP_006481585.1	43
COP9 signalosome complex subunit 5a isoform X1	XP_006450164.1	31
Copper transporter 1	XP_006424955.1	1
Protein SRC2-like	XP_015389034.1	5
Polyubiquitin 11	XP_006480632.1	1
Actin-depolymerizing factor	XP_006489736.1	1
Lectin	XP_006495232.1	1
Ran-binding protein 1 homolog a-like	XP_006488552.1	68
Lipid-transfer protein precursor	NP_001275802.1	23
Probable purine permease 11 isoform X2	XP_006464374.1	19
Pathogenesis-related protein 1-like	XP_006486822.1	30

Table S2. Oligonucleotides used in this study.

Oligonucleotide	Sequence	Use
HrpEf	ATCAGGATCCATGGAATTATTACCGCAAATCAG	Full HrpE cloning in pET32a
HrpEr	ATACAAGCTTTTACTGGCCAACGAGCTGCTT	Full HrpE cloning in pET32a
NHrpEr	ATACAAGCTTTTACGAGGCGTTCATGTCACTG	N-terminal HrpE cloning in pET32a
CHrpEf	CAGTAGAATTCGACGAGGCCCAGAAGTCCATG	C-terminal HrpE cloning in pET32a
CsGRPf	CACGTGTCGACATGGGTTCCAAATTGTT	Full CsGRP cloning in pGEX-4T3
CsGRPr	CACGTGCGGCCGCTCAGTTTTGAGGCT	Full CsGRP cloning in pGEX-4T3
HrpE-BDf	CAGTAGAATTCATGGAATTATTACCGCAAATCAG	Full HrpE cloning in pOBD
HrpE-BDr	CAGTACTGCAGTTACTGGCCAACGAGCTGCTT	Full HrpE cloning in pOBD
N-HrpE-BDr	CAGTACTGCAGTTACGAGGCGTTCATGTCACTG	N-terminal HrpE cloning in pOBD
C-HrpE-BDf	CAGTAGAATTCGACGAGGCCCAGAAGTCCATG	C-terminal HrpE cloning in pOBD
CsGRP-ADf	CAGTAGAATTCATGGGTTCCAAATTGTTCC	Full CsGRP cloning in pOAD
CsGRP-ADr	CAGTAGGATCCTCAGTTTTGAGGCTCGGTTTC	Full CsGRP cloning in pOAD
C-CsGRPADf	AGTAGAATTCGGTGGCCGTGGAGGTTAT	C-terminal CsGRP cloning in pOAD
AtGRP-3ADf	AGTAGAATTCGCATCTTCTGCCACAGTG	Full AtGRP-3 cloning in pOAD
AtGRP-3ADr	CAGTACTGCAGTTAGTGACCGGGCTGAGTC	Full AtGRP-3 cloning in pOAD
C-AtGRP-3ADf	AGTAGAATTCGGCTACGGTGACAATGGAG	C-terminal CsGRP cloning in pOAD
HrpEBiFCf	ATACAAGCTTGATGGAATTATTACCGCAAATC	Full HrpE cloning in pSAT
HrpEBiFCr	ATCAGGATCCCCTGGCCAACGAGCTGCTTGGC	Full HrpE cloning in pSAT
CsGRPBiFCf	ATACAAGCTTGATGGGTTCCAAATTGTTCCTTC	Full CsGRP cloning in pSAT
CsGRPBiFCr	ATCAGGATCCCGTTTTGAGGCTCGGTTTCAGG	Full CsGRP cloning in pSAT
CsACT-L	GAGCTGAAAGATTCCGTTGC	RT-qPCR (citrus housekeeping)
CsACT-R	TTGATCTTCATGCTGCTTGG	RT-qPCR (citrus housekeeping)
CsGST-L	AACCTACTTGGAAACACACTAGAAGA	RT-qPCR
CsGST-R	GTTCATCAGATATCTTAAGGCTGGTA	RT-qPCR
CsSOD-L	CAGAAGCATCACCAGGCTTA	RT-qPCR
CsSOD-R	CAATGCTTCCAGAGAACCAA	RT-qPCR
CsMAPK3-L	TTACATGATGAAGCCGATGAAC	RT-qPCR
CsMAPK3-R	TGAGTGCTAATGCCTCCTGATA	RT-qPCR
CsMKK4-L	GGCACCCTCGATACTTTGTT	RT-qPCR
CsMKK4-R	TAATTCCCTCCGTAGGCATC	RT-qPCR
CsPR1-L	AAAGTTGTTCAAACTTTTTGTCCTT	RT-qPCR
CsPR1-R	ACATGATCAATAGTAGGGATGTTAGC	RT-qPCR
CsPR4-L	GTGTGATTCTGTCACTTTGTCTACTG	RT-qPCR
CsPR4-R	ACTGTTTGTGACCCTTAAGCAC	RT-qPCR
CsHMGR-L	AAACTAATGTGGCTACACTGGTAGAG	RT-qPCR
CsHMGR-R	AATATGTAGATCCTTCCCATCATTTA	RT-qPCR
CsPAL-L	ATAATGGAACATATCTTGGATGGTAG	RT-qPCR
CsPAL-R	CTTGAATTATCCATAGAGACACCAAT	RT-qPCR
SlRpl2-L	CGTGGTGTTGCTATGAATCC	RT-qPCR (tomato housekeeping)
SlRpl2-R	GTCAGCTTTGGCAGCAGTAG	RT-qPCR (tomato housekeeping)
SlPti5-L	GCTAGACATGGTGCGAGAGT	RT-qPCR
SlPti5-R	TCTACTGAAACAGAGGCGTTC	RT-qPCR
SlLrr2-L	AAGATTGGAGGTTGCCATTGGAGC	RT-qPCR
SlLrr22-R	ATCGCGATGAATGATCGGTGGAGT	RT-qPCR
SlGras2-L	ATGTGAGGGCATGGAAAGAG	RT-qPCR
SlGras2-R	TCCATCCCAACAACATAGCTC	RT-qPCR
SlWRKY28-L	ACAGATGCAGCTACCTCATCCTCA	RT-qPCR

SlWRKY28-R	GTGCTCAAAGCCTCATGGTTCTTG	RT-qPCR
AtTUB-L	GAGAATGCTGATGAGTGCATGG	RT-qPCR (Arabidopsis housekeeping)
AtTUB-R	CAGGGAACCTCAGACAGCAAGT	RT-qPCR (Arabidopsis housekeeping)
AtPR1-L	TTCTTCCCTCGAAAGCTCA	RT-qPCR
AtPR1-R	AAGGCCCACCAGAGTGTA	RT-qPCR
AtGST1-L	ACCGTTGTTGAAGAAGAAGA	RT-qPCR
AtGST1-R	GCTCGTCGAAGAGTTTCTTA	RT-qPCR
AtPAL1-L	GAAGTTCTTCGAAAGCGTTA	RT-qPCR
AtPAL1-R	CCTCACTAAATCCTTTGCAC	RT-qPCR
CsGRP-L	CACGTGTCGACATGGGTTCCAAATTGTT	RT-qPCR
CsGRP-R	CACGTGCGGCCGCTCAGTTTTGAGGCT	RT-qPCR
AtGRP-3L	GTGGGCGACAAGGAGGAG	RT-qPCR
AtGRP-3R	TTAGTGACCGGGCTGAGTC	RT-qPCR
